# Examination for Meat-Inspector, February 5, 1901

**Published:** 1901-01

**Authors:** 


### EXAMINATION FOR MEAT-INSPECTOR, FEBRUARY 5, 1901.

The United States Civil Service Commission announces that on February 5, 1901, an examination will be held in any city in the United States where postal free delivery has been established for the position of meat-inspector in the Department of Agriculture.

The examination will consist of the subjects mentioned below, which will be weighted as follows:

| Subjects. | Weight. |
|---|---|
| 1. Spelling (second grade) | 5 |
| 2. Arithmetic (second grade) | 5 |
| 3. Letter-writing (second grade) | 5 |
| 4. Penmanship | 5 |
| 5. Copying from plain copy (second grade) | 5 |
| 6. Veterinary anatomy and physiology | 10 |
| 7. Veterinary pathology | 25 |
| 8. Meat-inspection | 40 |
| Total | 100 |

Information relative to the scope of the examination may be found in sections 42 and 65 of the *Manual of Examinations*, revised to January 1, 1900.

Applicants for this examination must be graduates of veterinary colleges. Age limit, twenty years or over.

The examination is held to establish an eligible register for the position of meat-inspector, for the reason that the present register is not ample to meet the needs of the service.

The examination is open to all citizens of the United States who comply with the requirements and desire to enter the service. All such persons are invited to apply, and applicants will be examined, graded, and certified with entire impartiality and wholly without regard to any consideration save their ability as shown by the grade attained in the examination.

Persons who desire to compete should at once apply to the United States Civil Service Commission, Washington, D. C., for application forms 304 and 375, which should be properly executed and promptly filed with the Commission.

